# Gastric Dilatation Masquerading as Anterolateral ST-Elevation Myocardial Infarction

**DOI:** 10.7759/cureus.41442

**Published:** 2023-07-06

**Authors:** Yuval Avidan, Vsevolod Tabachnikov, Amir Aker

**Affiliations:** 1 Cardiology, Carmel Medical Center, Haifa, ISR

**Keywords:** bariatric surgery, bedside echocardiography, acute gastric dilatation, ecg abnormalities, stemi mimickers

## Abstract

A variety of noncardiac conditions mimic the electrocardiographic changes of ST-elevation myocardial infarction (STEMI). Therefore, a physician must maintain a high index of suspicion when evaluating ST-segment elevation (STE). We present a case of epigastric pain secondary to ileus and gastric dilatation masquerading as anterolateral STEMI on an electrocardiogram (ECG). The STE promptly resolved following laparotomy. To the best of our knowledge, this is the first case of anterolateral STE secondary to gastric dilatation.

## Introduction

ST-elevation myocardial infarction (STEMI) is a medical emergency in which an electrocardiogram (ECG) is a central element in establishing its diagnosis, in conjunction with clinical presentation and cardiac biomarkers. ST-segment elevation (STE) is an important ischemia sign justifying the need for emergent coronary revascularization [1]. Although STE is the hallmark of STEMI, a variety of cardiac and noncardiac conditions are known to mimic STEMI on ECG. Reported abdominal pathologies include acute cholecystitis, acute pancreatitis, acute appendicitis, esophageal perforation, perforated duodenal ulcer, and stomach distention [2,3].

## Case presentation

**Figure 1:**
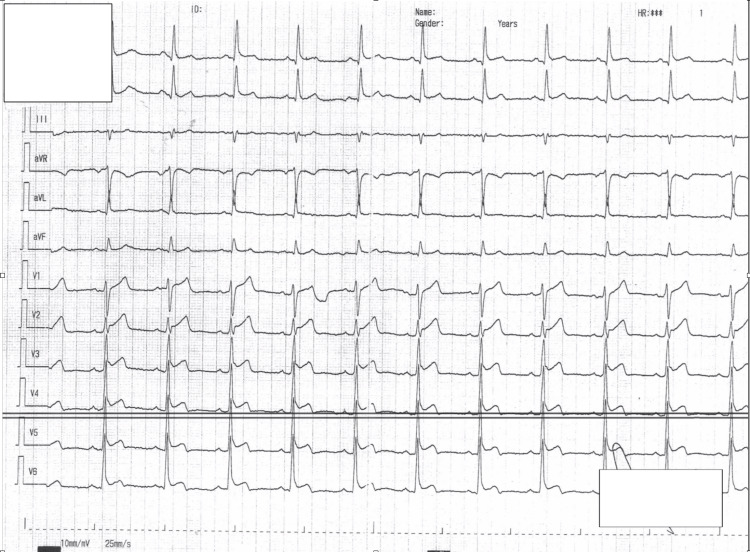
A 12-lead electrocardiogram recorded during the admission and demonstrating a normal sinus rhythm with ST-segment elevation in leads V1-V6, aVL, I, and II. aVL: augmented vector left

We present a male patient in his mid-50s who presented to our emergency department with an acute onset of severe epigastric pain, accompanied by nausea and sweating. He had eaten a large meal of hummus an hour prior to the onset of symptoms. He had neither cardiovascular history nor known modifiable risk factors besides a prediabetic state. His medical record included vertical banded gastroplasty surgery, open appendectomy, and post-operational ventral hernia, none of which was recent. His vitals and cardiac examination were unremarkable. His abdomen was distended with hypoactive bowel sounds and percussion tenderness. A 12-lead ECG showed anterolateral STE (Figure 1). It was interpreted by both the emergency physician and the computerized algorithm as STEMI.

**Figure 2:**
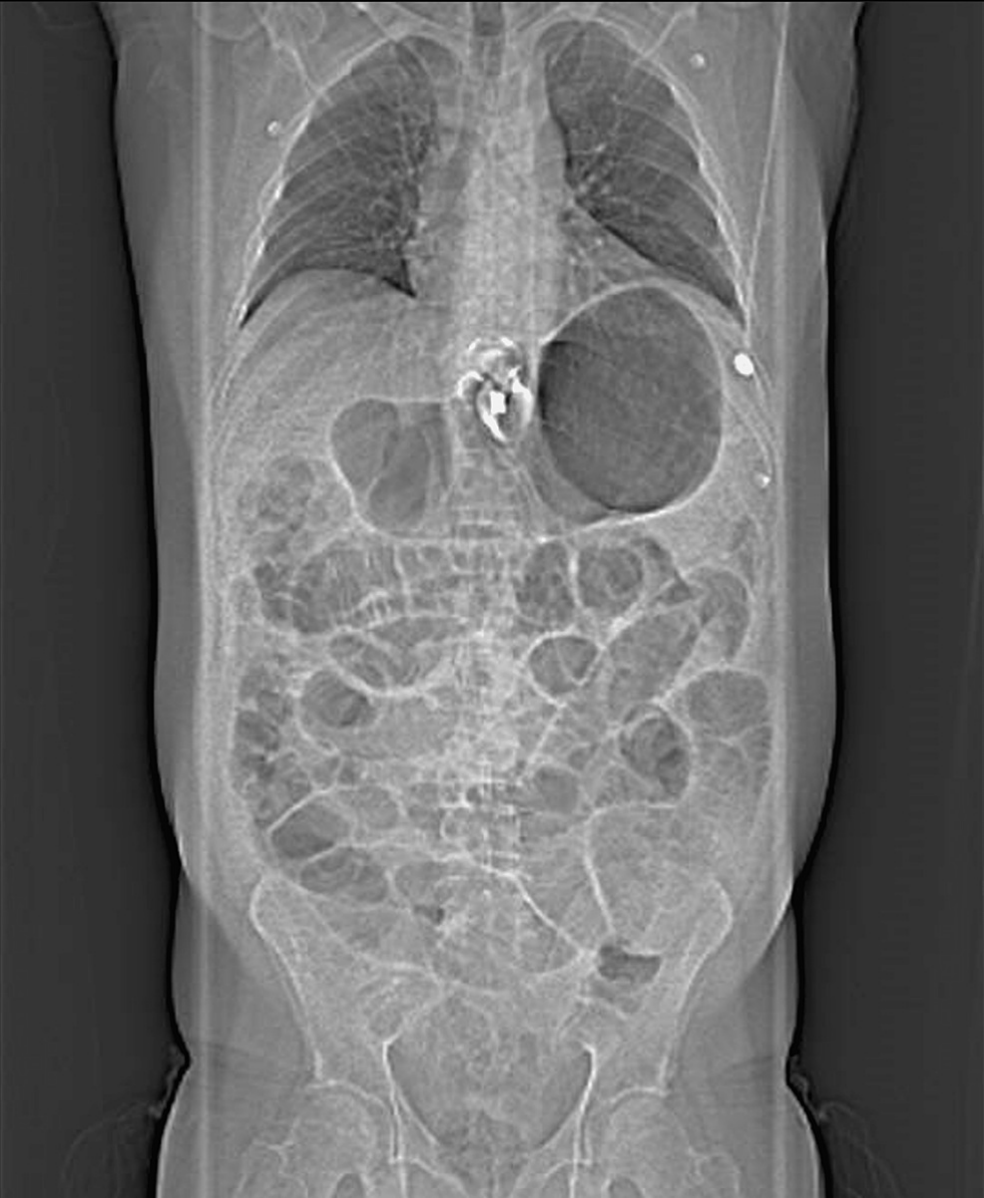
Computed tomography topogram showing marked gaseous distention of the stomach and dilated small bowel loops. Note the adjustable gastric band and the elevation of the left hemidiaphragm.

Next, emergent cardiology consultation was requested. At that point, anamnesis was fairly limited as the patient began vomiting and was in intense pain. Bedside echocardiogram showed good left ventricular conduction and no regional wall motion abnormalities. Due to the abovementioned supporting that the dominant clinical picture was extra-cardiac, we opted to pursue abdominal imaging (Figure 2).

**Figure 3:**
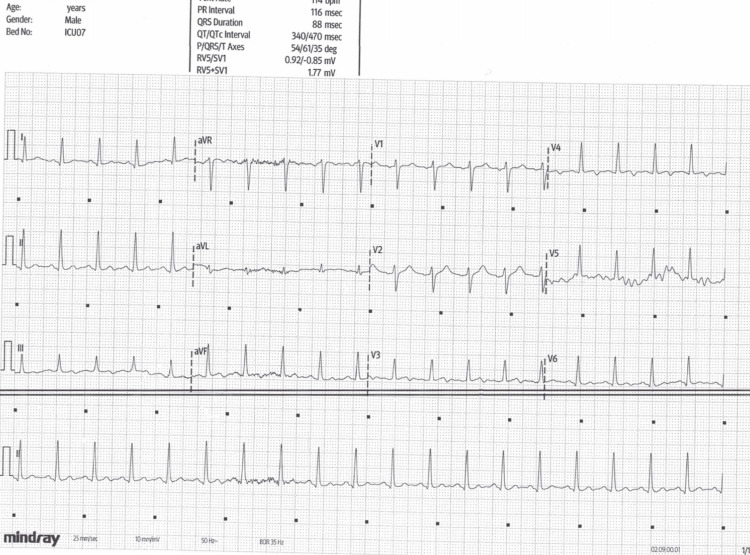
A 12-lead electrocardiogram recorded after the surgical intervention showing the resolution of the ST-segment elevation.

The computed tomography (CT) demonstrated small bowel obstruction with a markedly distended stomach. Subsequently, the laboratory analysis became available and revealed mild leukocytosis of 14.6 K/µL, serum amylase was 253 U/L, and liver enzymes, electrolytes, hemoglobin, and C-reactive protein (CRP) were within normal levels. Serial troponin I tests were negative (<13 ng/L). Nasogastric tube was inserted with prompt decompression along with fluid resuscitation. The patient underwent an emergent exploratory laparotomy with laparoscopic lysis of adhesions. The documented ECG changes promptly resolved following the operation (Figure 3). Transthoracic echocardiogram performed in the echocardiography unit demonstrated normal left ventricular contraction without segmental wall motion abnormalities, normal valvular function, and the absence of pericardial effusion. During the postoperative course, since there were no abnormal findings considering the ECG, continuous cardiac monitoring, and cardiac biomarkers, we opted for outpatient cardiac evaluation. The patient was seen in the outpatient clinic during three- and six-month follow-up with overall adequate recovery and no electrocardiographic abnormalities noted (the ECG is unavailable).

## Discussion

We present a case of a patient with anterolateral STE, which was initially thought to be cardiac in nature but turned out to be secondary to small bowel obstruction and marked gastric distention.

This case emphasizes one of the most important aspects of the emergency department, the correct triage in order to avoid diagnostic delay. The term “time is muscle” is used in cardiology to describe the value of rapid diagnosis and treatment in STEMI. However, even when physicians are confronted with supposedly “clear-cut” STEMI, they should keep an open mind and establish a list of potential differential diagnoses. Our case entails the combination between acute epigastric pain and ECG with STE in a young male. Precious time was wasted while waiting for the cardiology consult. In retrospect, greater attention to anamnesis and physical examination, together with the basic skills of bedside echocardiography, would have resulted in urgent surgery consult with faster abdominal imaging and operation.

As mentioned above, a variety of noncardiac pathologies have been described to mimic STEMI on ECG. In regard to our case, in 1965, Duke introduced the notion of ECG changes secondary to variation in diaphragmatic position with gastric dilatation, which resulted in a leftward shift of the QRS axis [4]. Other abnormalities have been described in the literature in similar settings, mostly consisting of T-wave inversions, ST-segment depression, and STE [2]. For instance, inferolateral STE was described with acute gastric distention [5,6]. The mechanism of such changes remains to be elucidated. Multiple hypotheses have been proposed, such as the compression of the diaphragmatic surface of the heart, electrolyte abnormalities, coronary vasospasm, fat embolism, myonecrosis from the release of pancreatic enzymes, vagal reflexes, and hypovolemia [7].

We suggest that the documented ECG changes were largely mediated via a direct effect of gastric distention on the heart, which possibly caused a relative change in the position of the heart with respect to the other intrathoracic organs. The anatomical distortion may have resulted in an alteration of the depolarization-repolarization course, which was manifested by the ECG in the form of STE.

Unlike previous cases that reported an inferolateral STE, a finding perhaps more easily rationalized by the direct pressure upon the inferior aspect of the heart, our case involved anterolateral STE. Possibly, these changes occurred secondary to his previous bariatric surgery. Indeed, prompt resolution of the ECG findings occurred following surgical intervention. Objective findings including echocardiogram and serial cardiac biomarkers demonstrated that there was no myocardial damage associated with these changes. The absence of a significant change in QRS axis in our patient would argue against major cardiac displacement. Moreover, the P-wave axis remained unchanged, and although a slight variation in the P-wave morphology was documented, this is most likely attributed to the sinus tachycardia.

## Conclusions

While the ECG remains an indispensable tool to diagnose acute myocardial infarction, physicians’ awareness to recognize acute abdominal conditions as potential mimickers is crucial to avoid diagnostic delay. This case highlights the value of meticulous anamnesis, physical examination, and basic knowledge of bedside echocardiography. Unlike previously reported cases, our patient had an anterolateral STE, perhaps due to the previous gastric banding.
